# Experiences of partners of professional nurses venting traumatic information

**DOI:** 10.4102/hsag.v23i0.1083

**Published:** 2018-08-13

**Authors:** Tinda Rabie, Melanie Wehner, Magdalena P. Koen

**Affiliations:** 1 Faculty of Health Sciences, North-West University, South Africa

## Abstract

**Background:**

Professional nurses employed in trauma units encounter numerous stressors in their practice environment. They use different strategies to cope with this stress, including venting traumatic information to their partners and other family members.

**Aims:**

To describe how partners of professional nurses cope with traumatic information being vented to them.

**Methods:**

A qualitative research method with an interpretive descriptive inquiry design was used to explore, interpret and describe the coping experiences of the nurses’ partners. Purposive sampling was used to select a total of 14 partners, but only ten participated in semi-structured interviews. Tesch’s eight steps of open coding were used for data analysis.

**Results:**

Four main themes were identified indicating adaptive and maladaptive coping skills, namely partners’ experiences of traumatic information vented to them; partners’ coping activities; reciprocal communication and relationship support between partners and nurses; and resilience of partners to deal with the nursing profession.

**Conclusion:**

Partners employed different ways to cope with traumatic information. It was essential for partners and nurses to be supported by nurses’ practice environments and to develop resilience to fulfil reciprocal supportive roles in their relationships.

## Introduction

Resilience is the human capacity to adapt, thrive and ‘maintain relatively stable, healthy levels of psychological functioning’ in response to potentially traumatic events (Bonanno 2004). This study explored and described how professional nurses’ partners coped with the venting traumatic information and how partners ‘bounced back’ from the vented traumatic information to fulfil a supportive role to the professional nurse.

Professional nurses daily confront different stressors in their practice environment such as conflicts with physicians, problems with peers and supervisors, discrimination, heavy workloads, long working hours, inadequate resources for performing their work productively, a shortage of equipment and supplies, dealing with difficult patients, traumatic situations, sickness, dying patients, death, and family members of critically ill or injured patients (Suresh, Matthews & Coyne 2013). Some specialised nursing fields such as trauma nursing, midwifery, intensive care and theatre nursing are reportedly more stressful and traumatic because of the highly stressed nature of these environments (Van der Wath, van Wyk & Janse van Rensburg 2013).

Trauma nurses must be able to think on their feet to save lives as the first line of contact when a patient arrives at the hospital (Oliveira et al. 2014). Professional nurses also see patients with broken bodies, broken souls or being severely traumatised. This situation requires professional nurses not only to deal with their primary responsibility of attending to the trauma patient but also to attend to the anxious family members (Oliveira et al. 2014; Van der Wath et al. 2013).

Professional nurses experience these events as being traumatic, causing feelings of helplessness (Komachi et al. 2012). When professional nurses are constantly exposed to such stressors, they might develop emotional or psychological disorders such as compassion fatigue, post-traumatic stress disorder, secondary traumatic stress (STS) and burnout (Özden, Karagöglu & Yildrim 2013). Consequently, professional nurses apply different techniques to cope with their work experiences, such as sharing their experiences with other colleagues and family members (Laal & Aliramaie 2010). However, these experiences are often shared with the nurse’s partner as being the first person with whom the nurses interact after work. Confiding in their partners makes the professional nurses feel safe and cared for (Laal & Aliramaie 2010), implying that partners share both pleasurable and traumatic aspects of nurses’ work-related experiences.

Venting, also known as social sharing, is recognised as one of the primary ways of coping and also of connecting with people (Nils & Rimé 2012). When venting to a partner with whom one has an emotional connection, the partner not only listens but also connects emotionally and sympathises with the person venting.

When professional nurses vent traumatic experiences, they also expose their partners to the traumatic events; this is known as secondary trauma. Secondary trauma can be described as behaviour and emotions resulting from knowledge about a traumatising event experienced by a significant other or by identifying with the experiences and feelings of a direct victim (in this study the professional nurses working in trauma units) (Holdsworth 2009). However, not much is known about the effect of this phenomenon on the professional nurses’ partners. According to Figley and Figley (2009), the partners might become empathetically involved, reliving the event with the professional nurses. This could cause the partners to be indirectly traumatised by the exchange of information, potentially affecting the partners’ own emotional and physical lives. The opposite might also be true; partners might be resilient and have the ability to cope with the vented information.

Couples who experience high stress levels might perceive their relationships as being less satisfactory (Neff & Karney 2007). However, some relationships might remain unharmed and become more resilient when facing stressors. Stressful events could afford both individuals, as well as the relationship, including the opportunity to improve their coping skills and resilience (Neff & Broady 2011). Relationships are an important source of satisfaction, happiness and general well-being (Koen & Du Plessis 2011; Tolpin et al. 2006). This could assist the professional nurses’ partners to use their coping skills to increase their confidence and strengthen their resilience as well as their relationships.

Coping is defined by Chesney et al. (2006) as a behavioural or cognitive effort to manage situations that are seen as stressful. Coping has also been defined as a response aimed at diminishing the physical, emotional and psychological burden associated with life’s stressful events (Tuncay et al. 2008). Therefore, coping is the process of interaction between a person and the situation depends on a person’s perceived ability to manage the stressor (Meehan, Peirson & Fridjhon 2007).

Coping is an active process comprising adaptive and maladaptive coping skills (White, Duncan & Baumle 2011). Adaptive coping skills deal with the stressors directly and imply a positive way of coping. Maladaptive coping skills are negative if the stressor is avoided, potentially preventing progress towards resolving the stressful situation (White et al. 2011).

It is important for partners to have good coping skills in order to support each other during stressful times. When a couple uses adaptive coping skills in their relationship and receive sufficient support from each other, their physiological stress will be reduced (Gunlicks-Stoessel & Powers 2009). The ultimate goal is for a couple to have stronger coping skills as individuals and as a couple, enabling them to cope better and to live life to the full, making them more resilient in themselves and in their relationship.

Laviola and Macri (2013) mention that the way in which people control their stressors directly affects whether they will build resilience. Building one’s adaptive coping skills will also contribute to increasing one’s resilience (Laviola & Macri 2013). Learning to cope with traumatic information will enable the partner to deal more effectively with the information, serving as an experience for coping with other stressful situations. Experience in dealing effectively with moderate-level stressors might protect partners against the potentially harmful effects of compounding emotions as a result of unresolved stressors (Updegraff & Taylor 2000). Dealing with stressors requires knowledge about using different strategies, having confidence in one’s ability to deal with events as a result of previous experience and knowing how to correctly evaluate potential threats (Updegraff & Taylor 2000), implying the use of coping skills to build resilience towards stress (Laviola & Macri 2013).

### Research problem statement

The nursing profession often involves a demanding, stressful and even a traumatic practice environment (Laal & Aliramaie 2010; Suresh et al. 2013). This is because of the different stressors and traumas to which professional nurses are exposed every day. These stressors include having to deal with difficult patients, sickness, dying patients, death, and traumatised family members (Komachi et al. 2012; McIntosh & Sheppy 2013). According to Adriaenssens, De Gucht and Maes (2015), this is even more true for nurses working in trauma units as they are confronted with more traumatic experiences such as mutilation and death. Professional nurses’ partners are often the nurses’ only support system when encountering stressors at work, and therefore nurses might vent traumatic information to their partners. Holdsworth (2009) found that when traumatic information is shared, the person listening (implying the professional nurses’ partners) in this study might experience feelings of being emotionally overwhelmed, which could potentially result in stress disorders such as secondary trauma, emotional fatigue and burnout. The researchers could not find studies about the coping experiences of professional nurses’ partners, to whom traumatic information has been vented.

### Purpose (aims) and objectives

The purpose of this study was to explore and describe the coping experiences of professional nurses’ partners to whom traumatic information has been vented.

### Definitions of key concepts

#### Partner

A partner is defined by the Merriam-Webster (2009) as a person with whom one shares a committed relationship. In this study a partner refers to a person in a relationship with a professional nurse, married or unmarried.

#### Venting

Venting refers to the urge felt by an individual to talk about his or her feelings or to share experiences with others (Rimé et al. 2010:1030).

#### Resilience

Resilience is the human capacity to adapt, thrive and ‘maintain relatively stable, healthy levels of psychological functioning’ in response to potentially traumatic events (Bonanno 2004:20).

## Materials and methods

### Design

A qualitative design with an interpretive descriptive approach (Thorne 2008) was used to explore, interpret and describe the coping experiences of partners of professional nurses venting traumatic information to them.

### Population

Partners of professional nurses working in the trauma units of two private hospitals in the Gauteng Province of South Africa were included in the study. All partners had to be in a relationship with a professional nurse for at least one year.

### Sampling and sample size

Purposive sampling was used and ten partners (*N* = 14; *n* = 10) agreed to participate. The partners were included if they were partners of professional nurses … practicing in a casualty unit in one of two participating private hospitals in Gauteng, able to communicate in English or Afrikaans and both the partners and the professional nurses … as being ‘couples’ in relationships for at least one year.

### Data collection procedure

Data were collected from April to June 2015. The researchers obtained permission from the ethical review board of the private hospital group to conduct the study. The interviewer gained entry to the hospitals by contacting the unit managers of the participating trauma units, who were the primary mediators. The mediator arranged with the professional nurses of each shift to meet the interviewer who explained the research project and handed out informed consent forms in sealed envelopes for the nurses’ partners. This implied that the professional nurses were the secondary mediators; they relayed the information and informed consent forms to their partners who could make their own decisions. After 7 days the interviewer collected the signed consent forms from the primary mediators and arranged a suitable date, time and venue for conducting the semi-structured individual interview with each participant. The interview schedule included the following questions: How long have you been in your current relationship? How did you cope in the beginning of your relationship with the professional nurse venting traumatic information? Has your way of coping changed over the years? If yes, how? Why do you think partners of professional nurses need to be resilient? What personal strengths do you have that enable you to be resilient? Do you think partners of professional nurses need to be more resilient than partners of other professions? What advice would you give other partners to help them to be more resilient?

### Data analysis and trustworthiness

The digital recordings were transcribed verbatim and coded with the assistance of a co-coder. Data analysis was performed by using Tesch’s eight steps of open coding (Creswell 2009). The findings of the analysed data were verified by the co-coder. Truth value was ensured by prolonged engagement of the studied population during the semi-structured individual interviews and data analysis. Applicability and consistency was applied by giving clear and precise descriptions of the research method to ensure the study could be repeated in a different context. Data collection was also continued when data saturation was obtained after interview number 7 as well as by using purposive sampling. These measures ensured that the findings of this study could be used as a guideline in other nursing contexts. Neutrality was ensured by not having personal bias or intentions, which could influence data collection, analysis and interpretation.

### Ethical considerations

The researchers respected the participants’ right to self-determination, their right to privacy, their right to anonymity and confidentiality, their right to fair treatment and their right to protection from discomfort and harm throughout the research.

## Results and findings

Four main themes with their sub-themes emerged from the data (see Table 1).

**TABLE 1:** Overview of the main themes and sub-themes.

Main themes	Sub-themes
Partners’ experiences of traumatic information vented to them	Partners’ primary experience of the shared traumatic information: Shocked Don’t pay attention Enjoyable, interesting and exciting Secondary experience and behaviour of shared traumatic information: Desensitised Adapted to become more resilient
Partners’ coping activities	Physical activities Detachment Research about information shared Personality traits Personal control and boundaries Compartmentalise
Reciprocal communication and relationship support between partners and nurses	Effective communication Relationship support: Emotional support Interpersonal relationship support
Resilience of partners to deal with the nursing profession	Lack of support between nursing colleagues Nursing occupation is more traumatic and stressful than other professions

### Theme 1: Partners’ experiences of traumatic information vented to them

Two sub-themes emerged as contributors to partners’ experiences of traumatic information being vented to them: partners’ primary experiences and secondary experiences and reactions to shared traumatic information.

The participants’ primary experiences included feelings of being shocked (five reports); not paying attention to the information (one report); and finding the information enjoyable, interesting and exciting (four reports) as denoted by the following quotes.

‘… in the beginning I was more shocked about the things she told me ….’ (Participant F, male)

‘… so I didn’t give a lot of attention to the information …’ (Participant E, male)

‘Well at first I found it a bit exciting actually. I enjoyed all the stories she would tell.’ (Participant H, male)

Initial response to traumatic information is shock (Tolpin et al. 2006). Collins and Long (2003) mentioned that some people who do not cope with traumatic information being shared with them, might attempt to protect themselves by ignoring this information. However, the research by Davis and McLeod (2003) revealed that some people craved sensational information, explaining why some partners might find the shared traumatic information as being enjoyable, interesting and exciting.

The secondary experiences and reactions to shared traumatic information included coping, by becoming desensitised (three reports) and adapting to becoming more resilient (three reports):

‘I got more and more desensitised to what she [professional nurse] would tell me.’ (Participant G, male)

‘… actually dating a nurse you become more resilient in life.’ (Participant F, male)

Persons exposed to traumatic information over a prolonged period of time could become desensitised to the information as a coping strategy (Foa & Hearst-Ikenda 1996). Neff and Broadly (2011) found that people exposed to moderate stressors, as the partners of professional nurses, gained the ability to become more resilient and they could adapt their views of the stressors to provide informative understanding. Turner (2014) maintained that adaptation of a person to change could be either positive or negative. Partners of professional nurses, who participated in the current study, adapted positively by becoming more resilient.

### Theme 2: Partners’ coping activities

Partners’ coping activities were evident as they engaged in different coping activities. These activities included physical activities (five reports); detachment (one report); research about information shared by the nurse (two reports); certain personality traits such as having openness (one report); having a strong personality (three reports); being analytical (two reports); and being practical (one report). Some participants mentioned personal control and boundaries (three reports) and lastly compartmentalising of their feelings (one report). The following quotes represent this theme:

‘It helps to walk and get some of my own frustration out; away from life and work and all that but also outside is a calming place. I find nature to be a very good way to connect and de-stress.’ (Participant F, male)

‘I don’t get emotionally involved in the details, and I detach from the information she gives me and rather deal with her and her emotions …’ (Participant H, male)

‘… to have strongest personality trait would be to … compartmentalise different parts of your life …’ (Participant D, male)

‘So the first thing I did was set boundaries.’ (Participant D, male)

‘I have had that interest and would read up about CPR and medical things …’ (Participant G, male)

‘I learned about what happens at her work and what needs to be done; by doing this I can now ask her more questions about her day and learn more.’ (Participant J, male)

‘… a strong personality handles the information better and easier.’ (Participant E, male)

Physical activity not only helps one to cope more effectively with daily stressors, but it also promotes one’s physical and mental health (Childs & de Wit 2014). In addition people could use adaptive and maladaptive coping skills in stressful situations, such as detachment, compartmentalising of feelings, boundary setting and gathering information about the traumatic event (Bolger 1990; Compas et al. 2001; Galor 2012; Gunlicks-Stoessel & Powers 2009; Skinner et al. 2003). Klimstra et al. (2013) supported the participants’ view that specific personality traits could enable one to cope with traumatic information or events.

### Theme 3: Reciprocal communication and relationship support between partners and nurses

Reciprocal communication and relationship support between partners and nurses were evident through effective communication and relationship support. Effective communication between the nurses’ partners and the professional nurses is a fundamental aspect (six reports) as illustrated by the following quotes:

‘And learn how to listen.’ (Participant G, male)

‘Good communication. Just open the line of communication.’ (Participant A, male)

‘… communicating is important …’ (Participant F, male)

It is important that there should be open communication and partners should learn how to listen to each other when they communicate to ensure a good relationship. Lavner, Karney and Bradbury (2016) agree with this statement by indicating that couples with effective communication have relatively satisfying relationships.

The participants emphasised the importance of relationship support in the form of emotional and interpersonal support when coping with stressful events as follows: partners would find a sense of belonging by giving the nurses emotional support (three reports); giving emotional support to the nurses helped their relationships (two reports); both partners must make an effort to spend time together (one report); and supporting the professional nurses by showing them affection (four reports). The following quotes illuminate this theme:

‘The fact that she is now giving [information] without me asking gives me a sense of purpose like I’m her protector.’ (Participant H, male)

‘… it helps the relationship to be understanding and supportive.’ (Participant A, male)

‘You got to have time for her, make time; they work different hours to what you do.’ (Participant I, male)

‘Um, ja, just show her you love her.’ (Participant J, male)

‘… or even just take her out, run her a bath; that way I did something to make it better for her …’ (Participant H, male)

Johnson (2002) reports that being in a relationship has many aspects that can make a partner feel fulfilled in and satisfied with the relationship. Research found that relationship satisfaction increases when partners feel that they have a definite role or a purpose to fulfil in the relationship. Literature also highlights that partners should plan to spend quality time, show mutual support to one another and show affection (Chapman 2015; Johnson 2002).

### Theme 4: Resilience of partners to deal with the nursing profession

Resilience of partners to deal with the nursing profession was developed as a result of a lack of support between nursing colleagues as the nursing profession involves more traumatic or stressful experiences than other professions.

The participants indicated that they needed to be more resilient in their relationships with the professional nurses. Many participants felt that the nurses lacked support in the practice environment between nursing colleagues and that they (the partners) had to fulfil this role (six reports) when they stated:

‘Yes, you are the one that they confide in and share events from work that they might not even share with colleagues …’(Participant A, male)

‘You see you are the only one some days that they can talk to …’ (Participant G, male)

‘… because partners of other professions don’t hear about horror stories that happened at the work of a nurse …’ (Participant B, male)

A lack of support from management and colleagues in the practice environment is an eminent stressor (De Wet & Du Plooy 2012; Delobelle et al. 2009; van Dyk 2007). This statement is supported by Rabie, Klopper and Coetzee (2017) who maintain that collegial support between nurses, nurses and physicians and nurses and their managers is important to establish and sustain a positive practice environment.

Nurses are believed to experience more stress than people in most other professions as a result of the nature of their profession (Van der Colff & Rothman 2014). Participants in this study unanimously agreed that the nursing profession is more traumatic and stressful than other professions. Therefore, nurses’ partners need to be more resilient because they are being exposed to traumatic information in their relationships with the professional nurses (10 reports):

‘Other professions don’t bring so much stories with them home …’ (Participant E, male)

‘Also it’s not like other professions; nurses work with real people and their lives so it’s much more life and death.’ (Participant G, male)

‘Yea, in a way a partner of any person in the medical profession that deals with guts and gore needs to have a stomach to deal with this.’ (Participant H, male)

Reasons why the nursing occupation has more stressors, include high workloads and staff shortages (Rabie, Coetzee & Klopper 2016). Other stressors include death and dying patients, feeling that one’s work is not valued, sleep deprivation, prolonged working hours, dealing with difficult patients, infectious and dangerous occupational risks and professional conflict between staff (Cheng, Tsui & Lam 2015).

## Discussion

Partner’s experiences concerning traumatic information shared with them differed. Under primary experiences some partners were shocked, did not pay attention, or found the information to be enjoyable, interesting and exciting. Concerning secondary experiences and reactions, some partners become desensitised, and others adapted by becoming more resilient, while others gained knowledge while they enjoyed listening to their partners. It is therefore important to focus on the partners’ initial reactions.

Professional nurses should be supported in their practice environment to develop effective coping skills. As a result of the support given to these nurses to develop effective coping skills, their partners will also be able to benefit from the support system offered to nurses. Partners without effective coping skills could adopt maladaptive coping skills such as failure to pay attention or avoiding to cope with the information. In the long term, such maladaptive coping strategies could negatively impact on the partners and their relationships. In the current study, nurses’ partners adopted different coping strategies which were physical in nature, or by resorting to detachment, doing research, by utilising their personality traits (openness in the relationship, having a strong personality, being analytical or practical) or taking personal control and establishing boundaries or compartmentalising problems. By building on these coping skills, the partners could develop or improve resilience and cope better with future stressful events.

The dynamics of their relationships influenced partners’ ways of coping with traumatic information. It was observed that partners with effective coping skills transferred these skills to their personal and work lives, enabling them to cope better with stressors. Lastly, it became evident that partners regarded it to be important to be resilient for the sake of the professional nurses. The reality of the daily stressors to which professional nurses are exposed was highlighted by the partners. The stressors to which professional nurses were exposed did not affect them alone, but also affected their partners with whom they shared this information. Sharing this information might make the partners less resilient as there was no support or debriefing sessions for partners to deal with such information. Professional nurses lacked support from their colleagues in their practice environment. As a result, nurses sought the support from their partners, who had to deal with the shared information responsibly. Professional nurses need to become more aware of the way in which they share their experiences with their partners in order to have a healthy relationship. The partners of professional nurses need to discover strategies that work effectively for them in their relationships to establish and maintain healthy long term relationships. Managers in the practice environment have a responsibility to establish counselling or debriefing sessions to assist nurses and their partners to cope effectively with traumatic information.

## Conclusions, limitations and recommendations for future research

### Conclusions

The study’s result indicates a lack of support services for nurses in their practice environment. The lack of support in the practice environment for nurses leads to partners of nurses not to be able to cope well, which could potentially lead to maladaptive coping skills. Therefore, it is important that adequate support services (such as counselling and debriefing sessions) should be available to nurses to enable them to cope with traumatic experiences in the practice environment. The lack of support in the practice environment causes nurses to vent their trauma-related experiences to their partners. This is, however, unfair towards partners who are now expected to support and debrief their nurse partners, when supportive measures should be taken by management in the practice environment to assist nurses to cope with traumatic experiences.

### Limitations

The following limitations were identified:

- The study was only conducted in two private hospitals’ trauma departments in the Gauteng Province of South Africa, thus limiting this study’s findings to these settings.
- Because only male participants volunteered to participate in the study, the viewpoints of female partners were not obtained.
- The researchers could not recruit the partners without supplying details of the study to the nurses working in the trauma units. Having the professional nurses as the second mediators could have introduced bias discouraging their partners to participate in the current study.
- The professional nurses who vented traumatic information to their partners were not interviewed and this could have added to the richness of the data.

### Recommendations for future research

Management can support nurses more by developing workshops or giving nurses the opportunity to attend workshops focusing on the development of effective coping skills. Nurses’ partners should also have the opportunity to attend these workshops.
